# Morphological Seed Traits Structure Relationships Between Biocrusts and Plant Emergence

**DOI:** 10.1002/ece3.71450

**Published:** 2025-06-01

**Authors:** J. Bacovcin, C. McIntyre, C. A. Havrilla

**Affiliations:** ^1^ Department of Forest and Rangeland Stewardship Colorado State University Fort Collins Colorado USA; ^2^ National Park Service, Chihuahuan Desert Network Tucson Arizona USA

## Abstract

Understanding abiotic and biotic drivers of plant emergence and community assembly is a central goal of plant ecology. In drylands, extreme temperatures and water and nutrient limitations play strong roles in determining plant recruitment patterns. Biological soil crusts (biocrusts) modify the physical soil environment in drylands by increasing soil stability, moisture, and nutrient cycling. As such, biocrusts can have substantial effects on plant emergence depending on plant origin, functional group, and biocrust characteristics. However, understanding of possible mechanisms underlying these variable effects remains limited. To explore the possible role of seed traits in determining biocrusts effects on plant emergence, we conducted a meta‐analysis of 321 published studies to examine relationships between seed traits (e.g., seed mass, shape, and presence of appendages) and emergence responses to biocrusts. Results showed that (1) morphological seed traits were important predictors of emergence responses to biocrusts. For example, (2) seed mass controlled the effects of biocrusts on the emergence of native and non‐native plant species. In native plant species, emergence of large‐seeded species was inhibited by biocrusts, while emergence of small‐seeded species was slightly increased. (3) Seed mass also interacted with biocrust community composition to control emergence. For example, cyanobacteria biocrusts had no effect on the emergence of small‐seeded species but had positive effects on medium‐ and large‐seeded species, while mixed biocrust communities containing lichens and mosses increased the emergence of small‐seeded species but decreased emergence of medium and large‐seeded species. Seed appendages also mediated biocrust effects on emergence with negative effects on non‐native species lacking appendages but neutral effects on native species overall. These results increase our fundamental understanding of the role of plant functional traits in structuring biotic interactions and have implications for understanding biocrust controls on plant recruitment and community assembly processes in drylands.

## Graphical abstract

We used meta‐analysis to explore the possible role of morphological seed traits (e.g., seed mass, shape, and presence of appendages) in determining biocrusts effects on plant emergence. Biocrust effects on emergence were mediated by seed morphology (i.e., seed size, appendages) in interaction with other biocrust and plant characteristics. These results increase our fundamental understanding of the role of plant functional traits in structuring biotic interactions and have implications for understanding controls on plant community assembly processes in drylands where biocrusts are present.

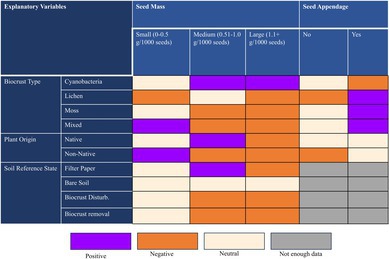

## 1 Introduction

Understanding the drivers of plant emergence and community assembly remains a consistent goal in plant ecology (Götzenberger et al. 2012; HilleRisLambers et al. 2012; Kraft et al. 2015). A number of abiotic and biotic factors can control which plant species germinate, establish, and persist in a given environment (Baskin and Baskin 2014; Belnap and Sharpe 1995; Kraft et al. 2015; Amat et al. 2015; Balazs et al. 2020). Abiotic factors such as climate and availability of key resources (i.e., soil moisture, nutrient availability, and space; HilleRisLambers et al. 2012; Carta et al. 2022) initially control which species can germinate and establish at a given site (Kraft et al. 2015). Biotic factors, including competitive and facilitative plant–plant interactions (e.g., Amat et al. 2015) and seed predation (Dylewski et al. 2020), also affect plant recruitment and resulting community composition. Plant–microbe interactions can also influence these processes (Bowker et al. 2022). Soil microbial communities can shape the physical soil environment by modifying the soil structure and the availability of resources important to plant emergence (Yang et al. 2016). As such, plant–soil microbial interactions can result in positive (Eldridge et al. 2021) and negative (Hoose et al. 2022) effects on plant emergence and recruitment depending on species and ecological context. For example, plant pathogens can infect dormant seeds and limit potential emergence (Hoose et al. 2022). Conversely, facilitative plant–microbe interactions (e.g., mycorrhizal fungi) can benefit plant recruitment (Trivedi et al. 2020).

Plant–microbe interactions may be particularly important for determining plant emergence and/or recruitment processes in resource‐limited environments such as Earth's arid and semiarid (dryland) regions (Mahmoudi et al. 2021) where water is the primary limitation of biotic processes (Naorem et al. 2023). Seeds require adequate levels of moisture, nutrients, and temperature to successfully germinate, which vary depending on plant species and ecological context (Baskin and Baskin 2014). In drylands, biological soil crusts (“biocrusts”)—interactions between the soil surface and photoautotrophic (e.g., cyanobacteria, algae, lichens, and bryophytes) and heterotrophic (e.g., bacteria, fungi, and archaea) organisms–reside on the surface and in the top millimeters of the soil (Weber et al. 2022) and mediate key ecosystem functions and soil biotic and physical conditions important to plant emergence (Lesica and Shelly 1992; Belnap and Gardner 1993; Havrilla et al. 2019). For example, biocrusts increase physical soil stability by exopolysaccharides, which function like glue, holding the soil together (Delgado‐Baquerizo et al. 2013) and provide protection against wind and water erosion. Biocrusts also influence soil hydrology, reducing water runoff and increasing moisture storage (West 1990; Eldridge et al. 2020). Biocrusts also mediate soil fertility, as they play a key role in the nitrogen (N) cycle and are responsible for nearly half of dryland N fixation (Barger et al. 2016). Soil temperature and surface albedo are also impacted by biocrusts. For example, dark pigmented biocrusts often increase soil surface temperature by decreasing soil surface albedo (Xiao and Bowker 2020). In addition to affecting soil abiotic processes, biocrusts may also influence emergence through direct biotic mechanisms. For example, microorganisms within biocrusts may assist in breaking seed dormancy of some plant species, possibly through signaling, nutrient release, and symbiotic associations (Eldridge et al. 2021). Given the physical and biological modifications of the soil environment, biocrusts have been shown to be important mediators of plant recruitment (Havrilla et al. 2019) and community assembly processes (Bowker et al. 2022; Song et al. 2022) in drylands.

A recent global meta‐analysis performed by Havrilla et al. (2019) found that biocrusts significantly affect dryland plant emergence, with variable effects based on the composition of the biocrust community (i.e., cyanobacteria, lichen, moss, or mixed dominated biocrust communities), plant origin (i.e., native versus non‐native to the study region), and plant functional group (e.g., C3 vs. C4 grasses). For example, overall, lichen‐dominated biocrusts decreased plant emergence, while other biocrust types did not affect emergence. Biocrust effects on plant emergence also differed across plant functional groups, with the strongest inhibition of emergence in C4 grasses. The study also found that biocrusts decreased the emergence of non‐native species but had overall neutral effects on native species.

One potential explanation for observed species‐specific and group‐specific effects of biocrusts on emergence could be the physical interactions between morphological seed traits (i.e., size, mass, shape, and structure) and the biocrust community (Havrilla et al. 2019; McIntyre et al. 2021; Zhang et al. 2016). Morphological seed traits, those traits describing the form and/or structure of seeds (Saatkamp et al. 2019; Carta et al. 2024), may be particularly important for determining outcomes of biocrust effects on emergence since seed structure can determine physical interactions with the soil surface (Baskin and Baskin 2014). For example, seed size or mass could control seed contact with the biocrust surface and associated resources. Seed mass is often positively associated with increased emergence on bare soil (Larson et al. 2015). Yet, this may not be the case with biocrusts. As the biocrust cover increases, seed contact with the mineral soil surface may decrease. Because biocrusts have complex microtopography, larger seeds may have limited contact with the mineral soil surface or favorable microsites within the biocrust surface. In contrast, smaller seeds may be more likely to fall into biocrust cracks (Havrilla and Barger 2018), increasing contact with the mineral soil surface and/or favorable microsites with greater shade and soil moisture and lower temperature. As such, we might predict that biocrusts may favor emergence of smaller seeded species. Seed appendages or awns, a bristle‐like extension from the lemma in the floret (Ntakirutimana et al. 2019), may also affect seed capture and positioning on the biocrust surface (Havrilla and Barger 2018). While seed morphological traits may provide a framework for understanding interspecific variation in emergence responses to biocrusts, seed traits have not yet been integrated into synthesis efforts exploring these interactions (Havrilla et al. 2019).

We conducted a quantitative meta‐analysis to explore the role of morphological seed traits in mediating biocrust effects on plant emergence. We integrated seed trait data from publicly available databases (i.e., TRY Plant database, Kattge et al. 2020), Kew Gardens Seed Information Database (SID) (Royal Botanic Gardens Kew 2020), and the Encyclopedia of Life (Parr et al. 2014) into a previously published global database of studies of biocrust effects on plant emergence (Havrilla et al. 2019). We then used meta‐analysis to explore the relationship among morphological seed traits (i.e., seed mass, shape, and the presence of seed appendage) and other ecological factors important for determining outcomes of biocrusts on plant emergence (i.e., biocrust type, plant functional group, and plant origin; Havrilla et al. 2019). We hypothesized that (1) morphological seed traits influence the effects of biocrusts on plant emergence. We posited that the emergence of larger seeds would be inhibited by biocrusts, possibly due to reduced contact with the mineral soil surface, while the emergence of smaller seeds would be increased due to access to resource‐rich microsites on the biocrust surface, and the emergence of seeds with appendages is inhibited by biocrusts due to physical interactions between biocrust microtopography and reduced contact of seeds with the mineral soil surface, while the emergence of seeds without appendages would be less affected. Second, we hypothesized that (2) seed traits mediate observed effects of biocrusts on the emergence of native vs. exotic plants and plants belonging to different plant functional groups (e.g., decreased emergence of non‐native plants and C4 grasses; Havrilla et al. 2019). For example, non‐native species might on average have larger seeds that are more likely to be accompanied by awns relative to native species (Tuthill et al. 2023), which could influence emergence outcomes in the presence of biocrusts. Results of our synthesis will support an improved understanding of the interactions between biocrusts and plant emergence and may be applied to better predict outcomes of plant recruitment and community assembly processes and ecological restoration across drylands.

## 2 Materials and Methods

### 2.1 Leveraging an Existing Published Database of Biocrust‐Plant Emergence Studies From Havrilla et al. (2019)

**FIGURE 1:**
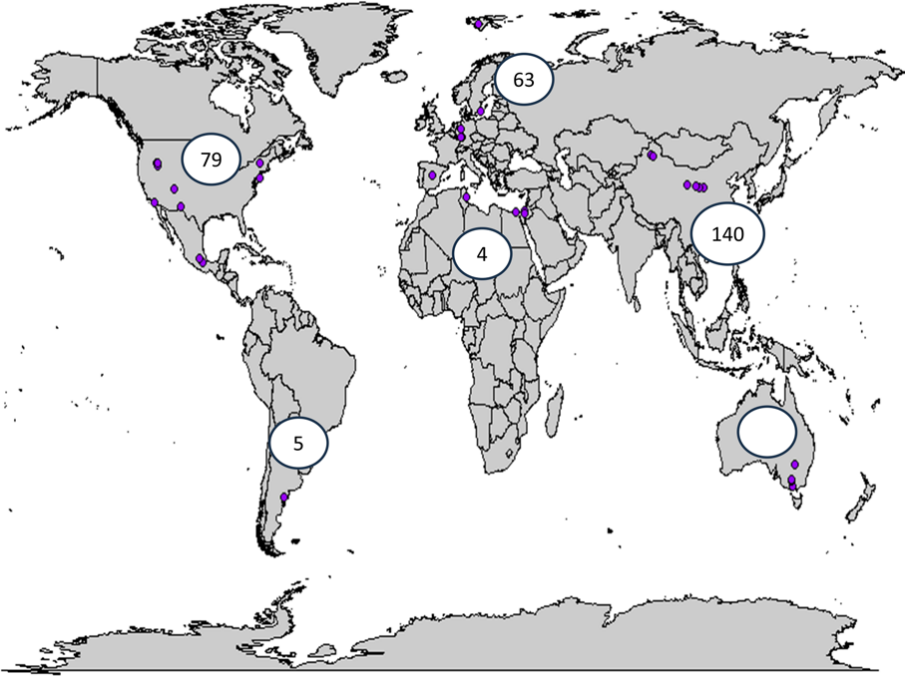
Map of locations of studies incorporated into the meta‐analysis.

We leveraged an existing, multilingual database of published literature on biocrust–plant interactions containing 491 unique comparisons (“studies”) of plant germination and emergence on biocrust versus controls lacking intact biocrusts (i.e., bare soil, disturbed biocrust, removed biocrust, or filter paper) across global drylands published by Havrilla et al. (2019), accessed in June 2021. The database includes emergence responses for 101 unique plant species across six continents (Figure 1) from literature published between 1940 and 2017. Using an existing data set and synthesis allowed us to more directly explore whether seed trait information can be used to understand patterns previously observed via a global meta‐analysis. While we acknowledge that additional studies on biocrust–emergence interactions have been published since 2017 (e.g., Bowker et al. 2022; Eldridge et al. 2021; Havrilla and Barger 2018; Hoose et al. 2022; Huber and Kollmann 2020; McIntyre et al. 2021; Slate et al. 2019; Song et al. 2020), we elected to use the (Havrilla et al. 2019) database due to its rigorous literature screening process and inclusion of studies identified with a multilingual search (as suggested in Zenni et al. 2023). As such, this data set and our synthesis can be seen as a study of a data set, but not an exhaustive synthesis of all available data published on this topic.

We extracted the following data from the Havrilla et al. (2019) database to construct the emergence data set used in our study: (1) plant species information (i.e., genus and species), (2) effect sizes showing the effect of biocrust on emergence relative to a control (i.e., log response ratio (LnRR; Equation 1)) (3) the estimate of within‐study variance (“ESTVAR3”; Equation 2; Hedges et al. 1999; Havrilla et al. 2019), and (4) metadata for all plant and biocrust covariates found to be predictive in the meta‐regression model for plant emergence responses to biocrust presence (i.e., BIOCRUST_TYPE, PLANT_FUNCTIONAL_GROUP, PLANT_ORIGIN, and SOIL_REFERENCE_STATE; described in Table 1; Havrilla et al. 2019).

(1) $$\ln=\left(X_{\text{crust}}/X_{\text{ctrl}}\right)$$

**TABLE 1:** Eleven‐candidate categorical predictor variables used within the mixed‐effects meta‐regression models.

Explanatory variable	Number of levels	Original (from Havrilla et al. 2019). or new variable	Description of variable levels	Selected variable	Included in the final meta‐regression model
BIOCRUST_TYPE	4	Original	Cyanobacteria, Moss, Lichen, Mixed; Classified by the dominant biocrust taxonomic group in the biocrust community as reported in the study. “Mixed” biocrust are communities containing substantial cover of both mosses and lichens.	Yes	Yes
PLANT_FUNCTIONAL_GROUP	7	Original	C3 grass, C4 grass, N‐fixing forb, Non‐N‐fixing forb, N‐fixing woody plant, Non‐N‐fixing woody plant, and Community; Plant functional group as designated in herbarium record for plant species. “Community” designates multiple plant species belonging to multiple plant functional groups.	Yes	Yes
PLANT_ORIGIN	3	Original	Native or non‐native; Corresponding to the native status of the plant in the study region. Non‐ Native species include any species not native to the study region	Yes	Yes
SOIL_REFERENCE_ STATE	4	Original	Bare soil, Biocrust removal, Biocrust disturbance, or Filter paper; Experimental control soil substrate for comparison to biocrust treatment as recorded in the study. “Biocrust removal” controls are those in which biocrust organisms have been removed from the soil surface while “biocrust disturbance” controls are those that have been mechanically disturbed or trampled.	Yes	Yes
SEED_MASS	3	New	Small, Medium, and Large; Originally collected as continuous variables but were binned into the three above based on seed mass. Breakdown was done in 0.5 g increments 0–0.5, 0.5–1, 1 + .	Yes	Yes
SEED_LENGTH	3	New	Short, medium, and long; Originally collected as a continuous variable but was binned into the three above categories based on seed length. Breakdown was done in 2 mm increments 0–2, 2–4, 4 + .	No	No
SEED_APPENDAGE	2	New	Yes or no; Corresponding to the presence or absence of an awn or appendage for any particular plant species.	Yes	Yes
SEED_APPENDAGE_LENGTH	3	New	Small, medium, and large; Originally collected as a continuous variable but was binned into the three above categories based on seed appendage length. Breakdown was done in 10 mm increments 0–10, 10–20, 20+	No	No
SEED_APPENDAGE_HYGROSCOPICITY	2	New	Yes or no; Corresponding to if the present awn is hygroscopic or not.	No	No
SEED_SHAPE	2	New	Linear or oval; A simplified classification of seed shape. Seeds that were roughly linear were assigned the linear category, seeds that were more rounded or elliptic were assigned oval.	No	No
SEED_MUCILAGINOUS	2	New	Yes or no; Corresponding to if the seed produces a mucilaginous coating or not.	No	No

Where *X*_crust_ is the mean plant response in the biocrust treatment, and *X*_ctrl_ is the mean plant response in the biocrust‐absent control.

(2) $$2=\left[{{\mathrm{SD}}^{2}}_{\text{crust}}/\left(n_{\text{crust}}\right)\left({X^{2}}_{\text{crust}}\right)\right]+\left[{{\mathrm{SD}}^{2}}_{\text{ctrl}}/\left(n_{\text{ctrl}}\right)\left({X^{2}}_{\text{ctrl}}\right)\right]$$

Where *X*_crust_ and *X*_ctrl_ are the mean plant response with and without biocrust, SD_crust_ and SD_ctrl_ are the standard deviation of the treatment and control means, and *n*_crust_ and *n*_ctrl_ are the number of replicates within biocrust and biocrust‐absent soil treatments.

### 2.2 Candidate Morphological Seed Trait Data

For each of the unique plant species contained in the Havrilla et al. (2019) database (*n* = 101 species), we compiled morphological seed trait data from publicly available plant trait databases: TRY Plant Database (Kattge et al. 2020), KEW Seed Information Database (SID) (Royal Botanic Gardens Kew 2020), and the Encyclopedia of Life (Parr et al. 2014). For a full description of our data collection process, see Data S1. From these databases, we extracted values for seven candidate morphological seed traits of interest (Table 1). Seed mass (g/1000 seeds) was selected to indicate a general measure of seed size and potential effects on emergence (Larson et al. 2015). A series of candidate traits reflecting seed architecture were also added to the database to explore potential interactions among seed physical structure and the biocrust surface to determine emergence outcomes. These included seed shape (i.e., ovate versus linear) and appendage presence (i.e., yes/no), appendage length (mm), appendage hygroscopicity (i.e., yes/no), and whether the seed has a mucilaginous seed coat (i.e., yes/no). Hygroscopic appendages are specialized seed structures that allow seeds to drill into the soil surface when the appendage is exposed to water (Elbaum et al. 2008). As such, we hypothesized that seeds with appendages may help seeds overcome physical barriers posed by biocrusts to emergence (e.g., soil surface hardness) and may increase emergence of these seeds on soils with biocrusts. Mucilaginous seed coats are an adaptation that takes the form of a mucilage layer when wetted (Yang et al. 2012). This mucilage coat is often adhesive and may aid with seed positioning and retention within favorable microsites and may provide lubricant for the plant radicle once emergence has occurred (Yang et al. 2012). Continuous variables (i.e., seed mass, seed length, and seed appendage length) were converted to categorical variables (Table 1) prior to data analysis to ease in interpretation of meta‐regression models.

### 2.3 Original Emergence Meta‐Regression Model From Havrilla et al. (2019)

The original emergence meta‐regression model from Havrilla et al. (2019) served as a base for our new model which incorporated morphological seed traits. Variables carried over from the original model were: BIOCRUST_TYPE, SOIL_REFERENCE_STATE, PLANT_FUNCTIONAL_GROUP, PLANT_ORIGIN, and STUDY_ID (Table 1). BIOCRUST_TYPE, SOIL_REFERENCE_STATE, PLANT_FUNCTIONAL_GROUP, and PLANT_ORIGIN, variables describing biocrust and plant characteristics, were included in the model as fixed effects. These variables were mainly populated with the information contained in study papers and assessments by Havrilla et al. (2019). For example, when the study paper did not report PLANT_ORIGIN of a given species the authors assessed plant origin by determining whether the plant species was native or naturalized to the region or continent in which the study was conducted using records in the USDA Plants Database (for North American studies) and/or the Kew SID. The residual between study variance (‘STUDY_ID’; Havrilla et al. 2019) for each unique study included as a random effect. This original meta‐regression model and all subsequent statistical analyses in this study were conducted in R (version 4.3.0; R Core Development Team 2021).

### 2.4 Preliminary Data Exploration and Candidate Morphological Seed Trait Variable Selection

To explore the relative importance of the candidate morphological seed trait moderators and their potential interactions with one another and original moderators from the Havrilla et al. (2019) model, we used a three‐step variable selection process. First, we examined the sample size of the various variables. This examination was used to inform if any variables needed to be excluded from the final model. Second, we explored potential correlations among candidate morphological seed trait predictor variables using correlation analysis via the *corrplot* package (Wei et al. 2017). If two variables had a correlation statistic *r* = +/−0.70, based on the cutoff levels suggested by Hinkle et al. (2003), then those variables could not be included in the same model. After reducing highly correlated variables down to a list of relatively orthogonal morphological seed traits, we used boosted regression tree (BRT) data exploration using the *gbm* package (Ridgeway et al. 2013), to explore the relative importance of the candidate moderators and their potential interactions in explaining variation among plant responses to biocrusts. Boosted regression tree analysis additively fits and combines multiple trees using a forward stepwise procedure, thus improving accuracy (De'Ath 2007). BRT analysis is ideal for complex data and unidentified distributions (De'Ath 2007), and additionally, can accommodate missing values in moderators (De'Ath 2007, Elith, Leathwick, & Hastie, 2008). We performed BRTs using the “gbm.step” function in the *gbm* (Ridgeway et al. 2013) and *dismo* packages (Hijmans, Phillips, Leathwick, and Elith 2017) as in Elith and Leathwick (2017). In each BRT model, we included only those moderators that had sufficient representation in the used dataset and corresponded to meaningful a priori hypotheses (Figure S1a); we then weighted each analysis according to the within‐study variance. Models were simplified using the “gbm.simplify()” function suggested by Elith and Leathwick (2017). Simplified BRT models for each analysis included the most influential moderators and ranked them according to their relative contributions (which are scaled to sum to 100% within each model—i.e., a particular moderator explains *X*% of the variation explained by the fitted BRT) to the explanation of variation in effect size. Relative variable influences were derived as an average of variable influence in all trees in each BRT model (Friedman and Meulman 2003). Potential interactions between moderators in final BRT models were explored using the “gbm.interaction()” function (Elith and Leathwick 2007). If BRT identified significant interactions among candidate predictor variables, we included these interactions in our initial meta‐regression models.

### 2.5 Mixed Effects Meta‐Regression

Following the selection of candidate moderators, meta‐analysis was performed by fitting meta‐regression models using the *metafor* package (Viechtbauer 2010) with restricted maximum likelihood estimation of parameters. We first used the *rma*() function to fit a pure random effects model to estimate the overall weighted mean effect size for the model (i.e., the weighted, overall log response ratio of the emergence of biocrust presence), with effect size weighted by within‐study variance and the residual between‐study variance component (‘STUDY_ID’) as a random‐effect. Then, we investigated the relative importance of the categorical fixed‐effect moderators (Table 1) included in the model by analyzing a series of mixed‐effect multiple meta‐regression models using the *rma.mv*() function, including a global model containing all the fixed factors (moderators) and candidate interaction terms being considered for that dataset and each of the nested subset models containing one more fixed factor. The model also contained the random effect STUDY_ID variable to account for residual between‐studies variation. When categorical moderators were significant (Q statistic < 0.05), differences in moderator levels were detected using planned contrasts with the *ghlt*() function from the multicomp package (Hothorn 2020). To explain residual heterogeneity and understand the potential effect of contextual factors on plant responses to biocrusts, we ran a series of separate univariate and bivariate interaction meta‐regression models for each analysis that included single significant moderators (Hoeksema et al. 2010; Havrilla et al. 2019). We chose to analyze the univariate and/or bivariate interaction models because this allowed us to maximize the number of studies that could be analyzed as not all the moderator variables were reported in every study. This also allowed us to maximize the studies used to calculate the intercept and slope or mean effect size values that described the relationship between each moderator variable and its effect on the log response of biocrust on emergence. This approach allowed us to calculate these values while still considering the effects of all moderator variables to ensure that each moderator variable analyzed still had a significant effect on LnRR in the presence of other moderators.

## 3 Results

### 3.1 Database Summary

Our final database contained 321 (64.6%) unique studies of plant emergence response to biocrust presence that were retained from the original 491 emergence studies within the original Havrilla et al. (2019) emergence database. Studies included in this integrated database spanned 12 different countries and all continents except Antarctica (Figure 1) and encapsulated the responses of 101 plant species from 27 families. Most (*n* = 248, 78.2%) were studies of emergence responses of native plant species, while 75 (21.8%) were of non‐native species. Studies contained a variety of biocrust community types: Cyanobacterial biocrusts made up 25.9% (*n* = 82), lichen made up 18.9% (*n* = 60). Data for all morphological seed traits were not available for all plant species. SEED_MASS (g/1000 seeds) was available for 314 (99.1%) studies, SEED_APPENDAGE (yes/no) was available for 250 (78.9%) studies, and SEED_SHAPE data was available for only 133 (42.0%) studies. SEED_APPENDAGE_LENGTH (mm) was accounted for in 60 (18.9%) studies. Seeds with hygroscopic awns accounted for only 2.50% of studies (*n* = 8) while the remaining 97.2% either lacked hygroscopic awns or lacked information on whether the species had hygroscopic awns (*n* = 308). Seeds with mucilaginous seeds accounted for only 7.60% of studies (*n* = 24) while most seeds studied were not mucilaginous (*n* = 292).

### 3.2 Candidate Variable Selection

From the correlation analysis, we determined that SEED_MASS and SEED_SHAPE were highly correlated (*r* = −0.71; Figure S1), and SEED_APPENDAGE and SEED_LENGTH were also highly correlated (*r* = +0.78; Figure S1). As such, we elected to retain SEED_MASS and SEED_APPENDAGE in our models since they were assumed to represent relative orthogonal morphological seed characteristics, there was a greater number of studies with this trait information available, and these traits were more directly relevant to our hypotheses. BRT data exploration showed SOIL_REFERENCE_STATE explained the greatest amount of variation in the responses of plant emergence to biocrusts 29.5%; (Figure S2), while BIOCRUST_TYPE explained 20.2% (Figure S2), PLANT_FUNCTIONAL_GROUP ~19.3% (Figure S2), SEED_MASS 17.0% (Figure S2), and finally SEED_APPENDAGE explained 10.4% (Figure S2) of variation. SEED_APPENDAGE_HYGROSCOPY and SEED_MUCILAGINOUS were not significantly influential. BRT analysis found that there were interactions between BIOCRUST_TYPE and SEED_MASS as well as SOIL_REFERENCE_STATE and SEED_MASS. The full final list of candidate variables included in the meta‐analysis can be found in Table 1.

## 4 Meta‐Analysis

### 4.1 Effects of Biocrusts and Key Moderators on Plant Emergence

**FIGURE 2:**
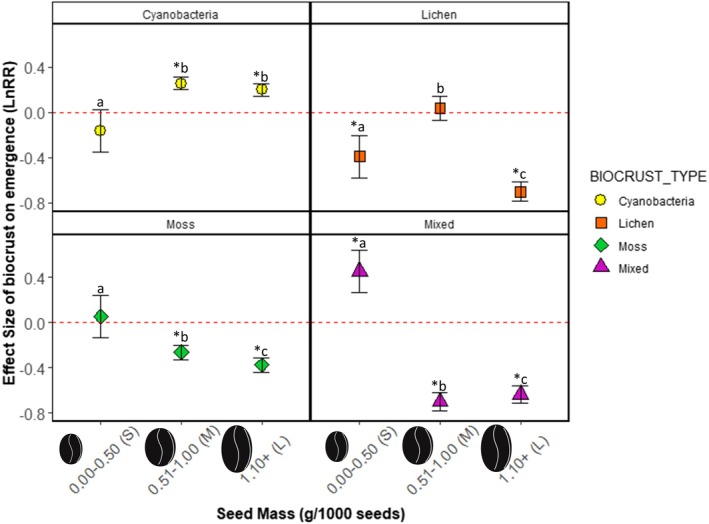
Effects of SEED_MASS on emergence responses to different biocrust types. Lowercase letters denote significantly significant pairwise differences (*p* < 0.05) within BIOCRUST_TYPE while “*” indicates significant differences from the red‐dashed line of no effect.

**FIGURE 3:**
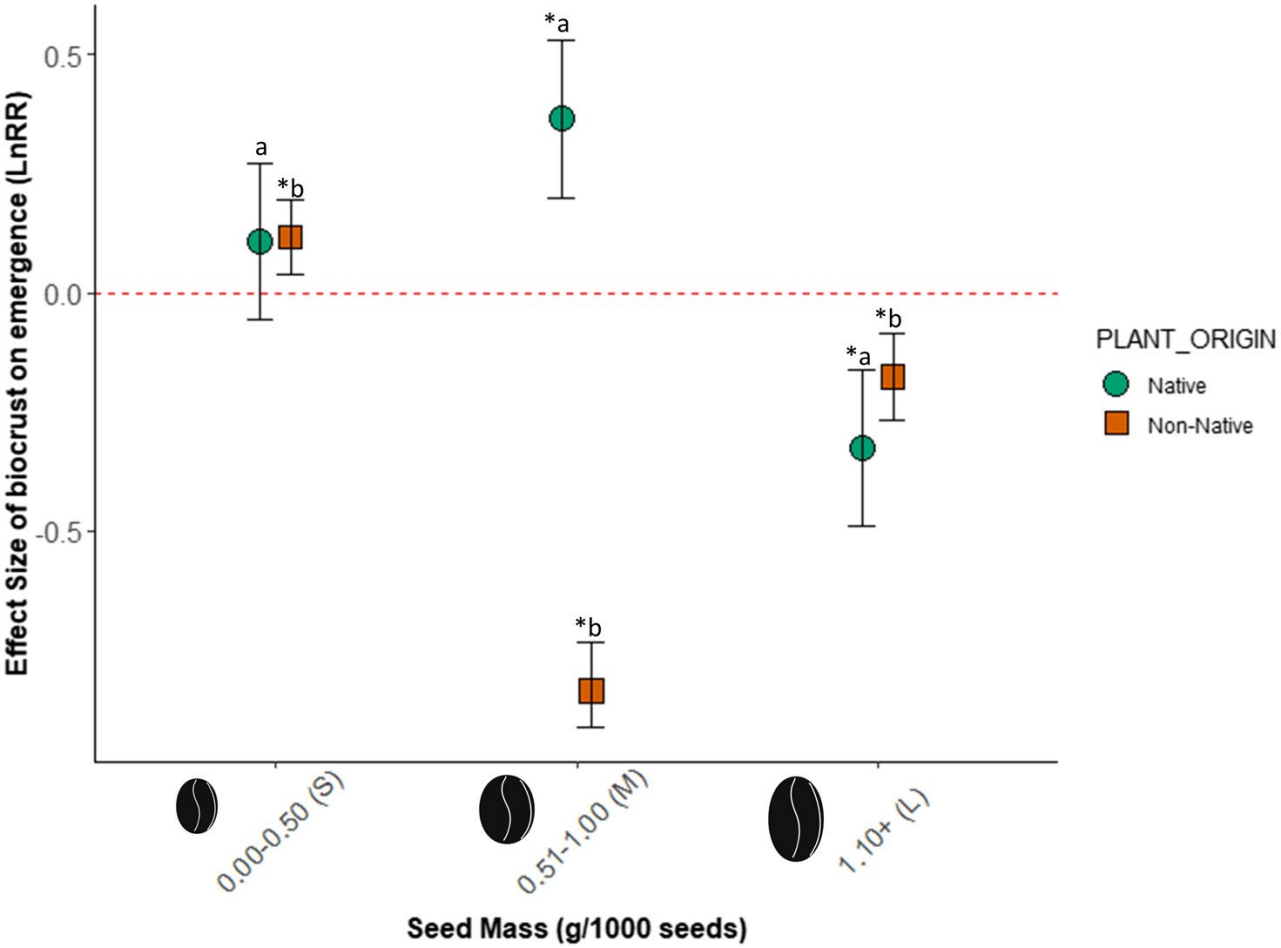
Effects of SEED_MASS on emergence responses to biocrust between native versus non‐native plant species (PLANT_ORIGIN). Lowercase letters denote significant pairwise differences among SEED_MASS *PLANT_ORIGIN levels while “*” indicates a significant change in emergence relative to the red‐dashed line of no effect.

**FIGURE 4:**
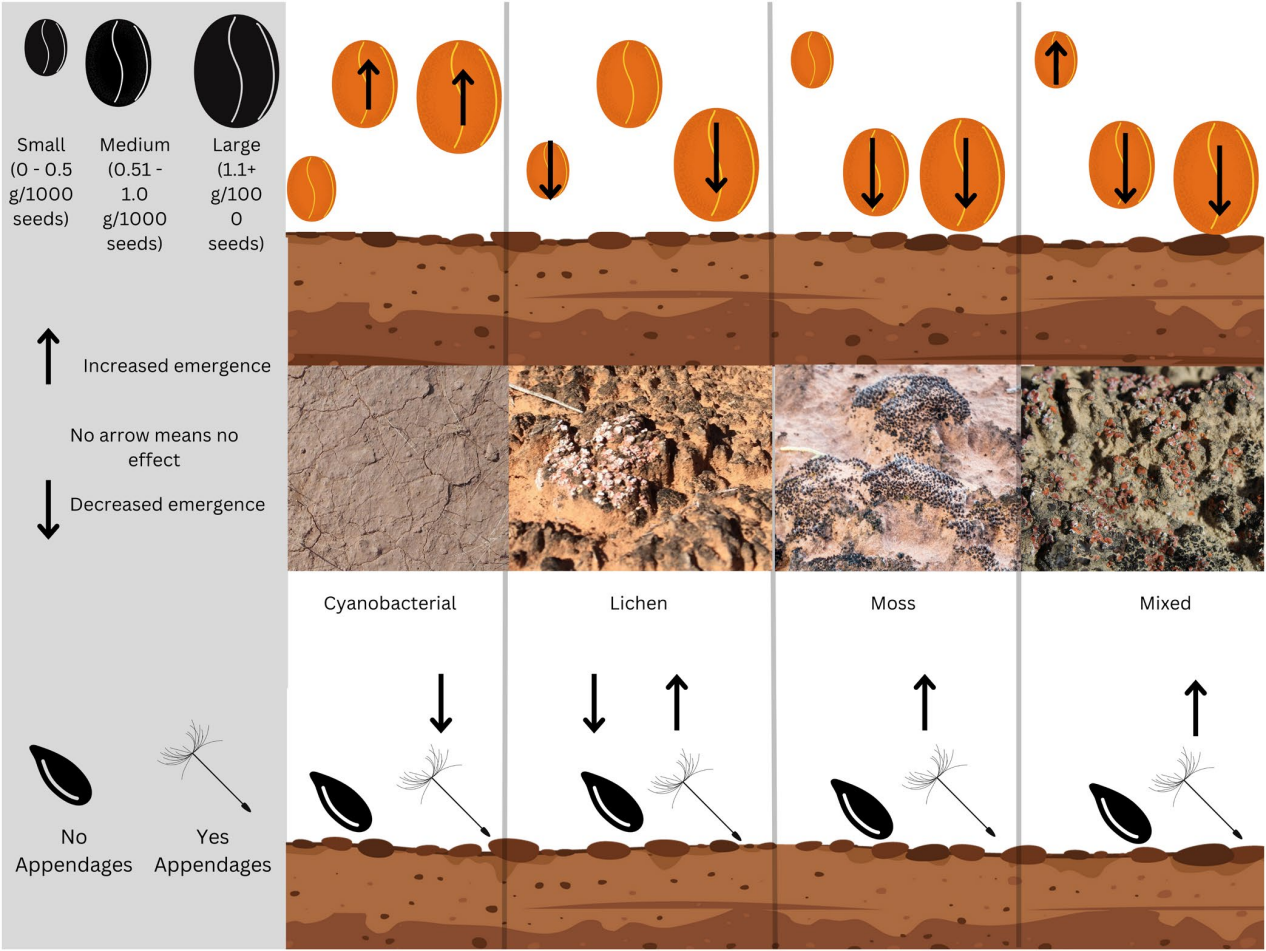
Conceptual diagram summarizing results for the overall direction of the effects of SEED_MASS (top) and SEED_APPENDAGE (bottom) on plant emergence responses to biocrust presence across different biocrust community types (i.e., cyanobacteria, lichen, moss, and mixed community types).

**FIGURE 5:**
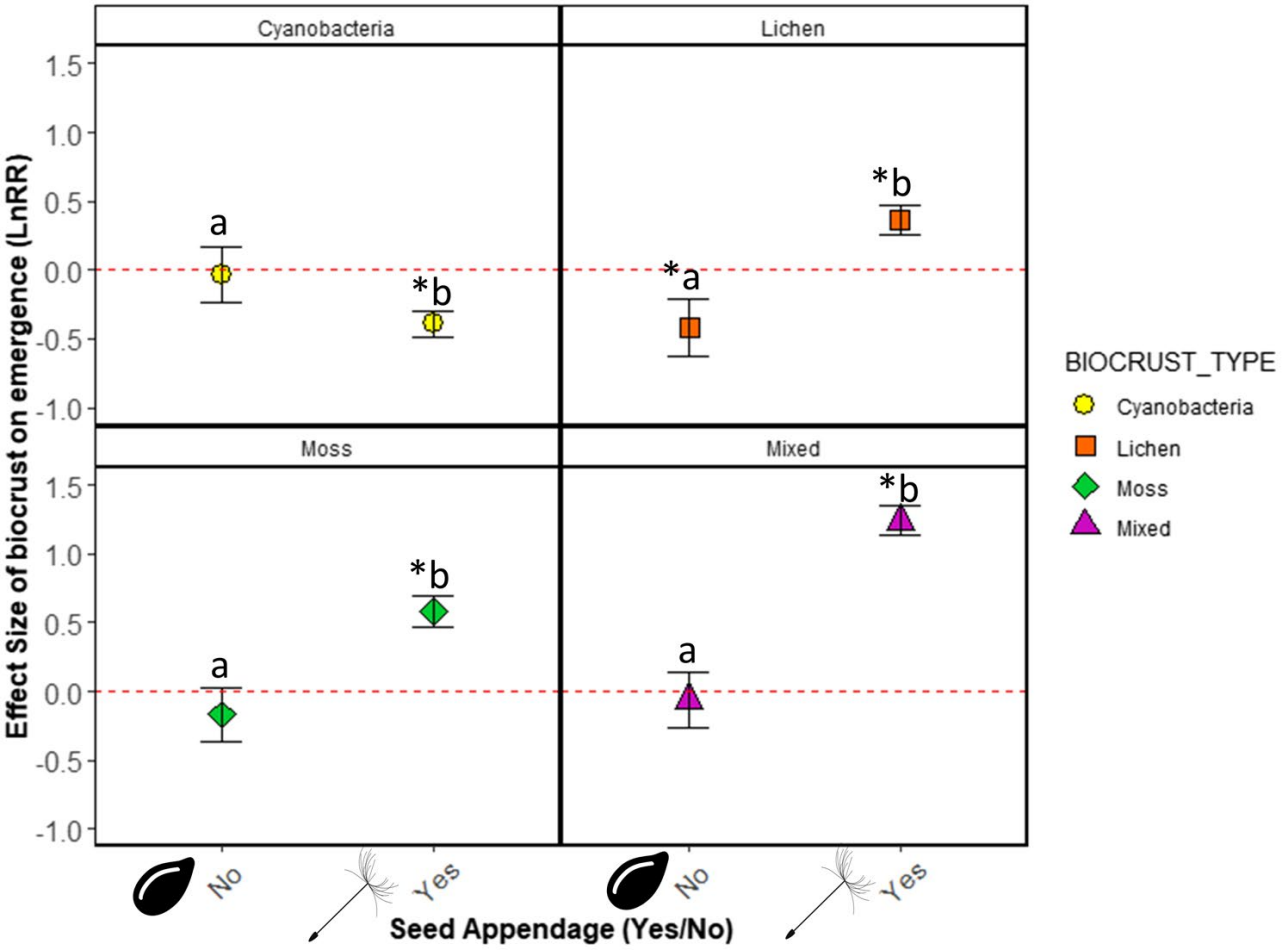
Effects of SEED_APPENDAGE on emergence responses to biocrust on different biocrust community types (BIOCRUST_TYPE). Lowercase letters denote significantly significant pairwise differences among SEED_APPENDAGE * BIOCRUST_TYPE levels while “*” indicates a significant change in plant emergence relative to the significant differences from the red‐dashed line of no effect.

**FIGURE 6:**
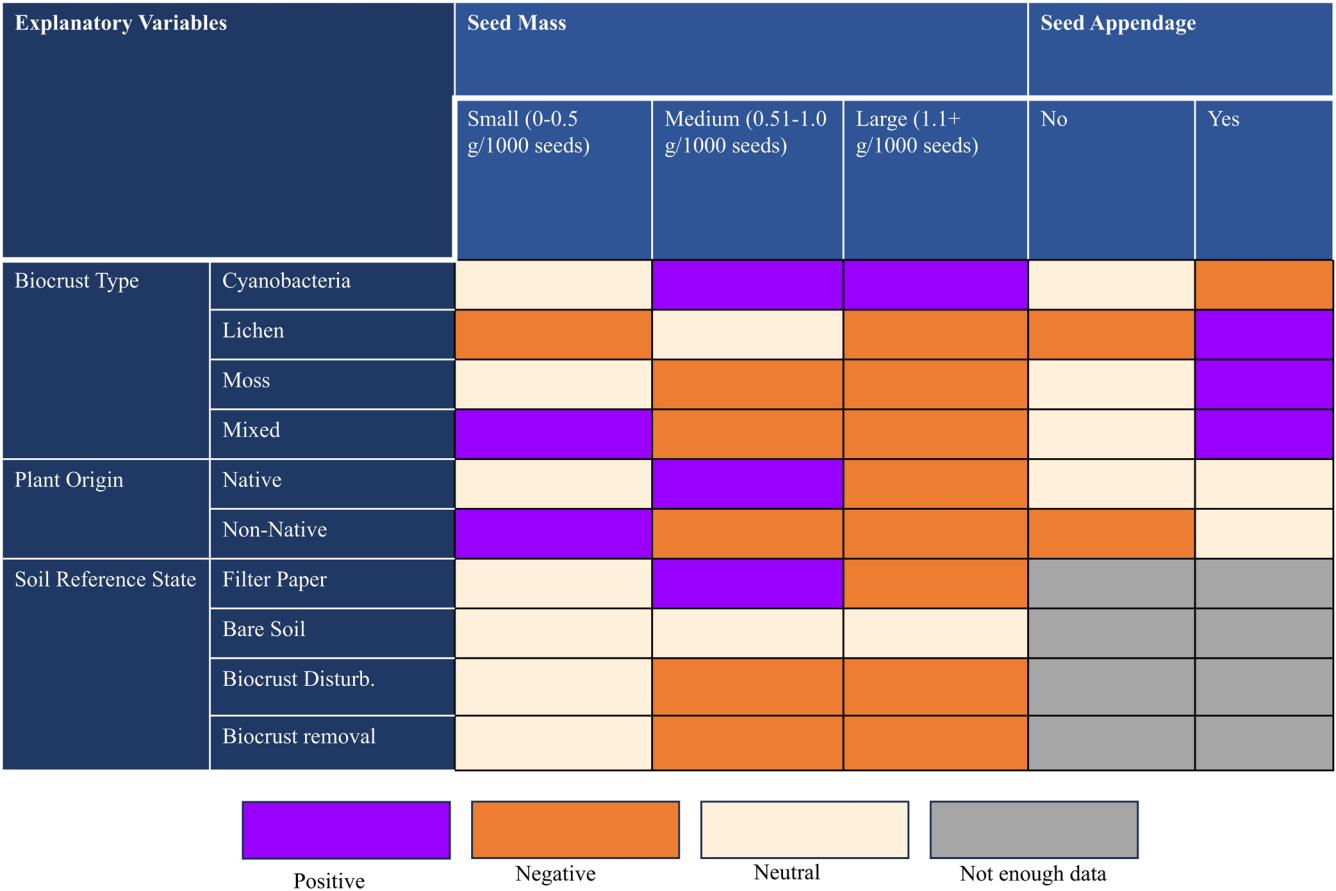
Summary diagram showing the effects of moderator variables SEED_MASS, SEED_APPENDAGE, BIOCRUST_TYPE, PLANT_ORIGIN, and SOIL_REFERENCE_STATE and their interactions on the effect of biocrust on plant emergence. Purple boxes denote a significant positive effect on emergence (*p* < 0.05), orange boxes denote a significant decrease in emergence (*p* < 0.05), beige boxes indicate that there was no significant influence on emergence, and gray boxes indicate that there was no analysis or not enough data to perform an analysis of the interaction.

Meta‐analysis showed that while there was no significant effect of biocrust on plant emergence overall (*p* = 0.444, Table S2), emergence responses varied depending on a variety of plant and biocrust characteristics including morphological seed traits (Figures 2, 3, 4, 5, 6). Each of the individual fixed effect variables (i.e., BIOCRUST_TYPE, PLANT_ORIGIN, SOIL_REFRENCE_STATE, PLANT_FUNCTIONAL_GROUP, SEED_MASS, and SEED_APPENDAGE; Table S2) significantly influenced plant emergence responses to biocrust and interacted with several other variables to determine the effects of biocrusts on emergence. For example, SEED_MASS moderated the effects of BIOCRUST_TYPE, PLANT_ORIGIN, and SOIL_REFERENCE_STATE on emergence, and SEED_APPENDAGE interacted with BIOCRUST_TYPE and PLANT_ORIGIN to influence the effects of biocrust on emergence (Table S2). Together, results from the meta‐regression model revealed overarching effects of moderators on the effect of biocrust on plant emergence.

First, as in Havrilla et al. (2019), we found different biocrust community types had differential effects on plant emergence overall. Relative to bare soil, cyanobacteria decreased plant emergence by 52.6% (Est = −0.840, *p* < 0.0001; Table S2), lichen decreased plant emergence by 320% (Est = −0.383, *p* < 0.001; Table S2), moss increased plant emergence by 39.3% (Est = 0.072, *p* = 0.033; Table S2), and mixed crusts increased emergence by 21.3% (Est = 0.009; *p* = 0.037; Table S2). Similarly, emergence responses to biocrust varied across different plant functional groups (PLANT_FUNCTIONAL_GROUP) and between native and exotic species (PLANT_ORIGIN). Overall, biocrusts increased the emergence of C3 grasses (+147%, Est = 0.262, *p* = 0.045), Nitrogen‐fixing woody plants (+490%, Est = −0.262; *p* < 0.0001) and Non‐nitrogen‐fixing woody plants (+21.5%, Est = −0.107, *p* = 0.013; Table S2), but decreased the emergence of C4 grasses (−310%, Est = 0.700; p = 0.045), Nitrogen‐fixing forbs (+190%, Est = −0.052; *p* < 0.0001), and Non‐nitrogen‐fixing forbs (−143.3%, Est = 0.094, *p* < 0.0001; Table S2).

PLANT_ORIGIN also played a role in determining emergence responses to biocrust presence. Overall, native species emergence was increased by 130% (Est = 0.735, *p* < 0.001; Table S2) and non‐native species had no effect on their emergence (Est = −0.176; *p* = 0.005, Table S2). SOIL_REFERENCE_STATE also influenced emergence responses; while there was no effect of intact biocrust on emergence relative to filter paper and disturbed biocrust controls (Est = −0.099, *p* = 0.772 and Est = 0.088, *p* = 0.123 respectively; Table S2), biocrusts increased emergence by 160% relative to bare soil (Est = 0.885, *p* < 0.001), and by 14.2% relative to biocrust removed controls (Est = 0.102, *p* = 0.029; Table S2).

SEED_MASS also affected emergence responses to biocrusts. Overall, small seeded (0.00–0.50 g/1000 seeds) and medium seeded (0.51–1.00 g/1000 seeds) species both experienced 280% increases on biocrusts respectively (Est = 1.46 and Est = 0.577, *p* < 0.0001 each, Table S2), whereas emergence of large seeded species (1.1+ g/1000 seeds) decreased 200% (Est = −0.247, *p* < 0.0001; Table S2).

Finally, SEED_APPENDAGE denoting the presence or absence of morphological appendages also influenced the effects of biocrusts on emergence. Overall, seeds with appendages experienced an over (530%) decrease in emergence on biocrust relative to controls (Est = −0.638, *p* < 0.0001; Table S2), while species lacking appendages experienced a more minor but significant decrease of 16.3% in emergence (Est = −0.640, *p* < 0.0001; Table S2).

#### 4.1.1 Seed Mass Interacts With Biocrust Community Type, Soil Reference State, and Plant Origin to Control the Effects of Biocrusts on Emergence

Seed mass also controlled the effects of biocrust community composition (i.e., cyanobacteria, lichen, moss, and mixed communities) on plant emergence. Overall, on cyanobacteria‐dominated biocrusts, medium and large‐seeded species experienced roughly doubled emergence in the presence of biocrust (Est = 0.253, *p* < 0.0001 and Est = 0.197, *p* < 0.0001 respectively; Figure 2, Table S2) while small‐seeded species were not significantly affected (Est = −0.167, *p* = 0.080; Figure 2, Table S2). On lichen‐dominated biocrusts, emergence of both small‐seeded (−35.7%; Est = −0.393, *p* < 0.0001; Figure 2, Table S2) and large‐seeded species decreased, with these effects greater for large‐seeded species, which experienced a 200% decrease in emergence in the presence of biocrust (Est = −0.701, *p* < 0.0001, Figure 2, Table S2). Emergence of medium‐seeded species was unaffected by lichen‐dominated biocrusts (Est = 0.0370, *p* = 0.487; Figure 2, Table S2). On moss‐dominated biocrusts, emergence of medium‐ and large‐seeded species was decreased by biocrust presence (−57.6%, Est = −0.263, *p* < 0.0001 and −125.1%, Est = −0.375, *p* < 0.0001 respectively; Figure 2, Table S2), while emergence of small‐seeded species was unaffected (Est = 0.0521, *p* = 0.580, Figure 2, Table S2). Finally, in mixed biocrust communities small‐seeded species experienced a nearly 400% increase in emergence (Est = 0.453, *p* < 0.0001, Figure 2, Table S2), while emergence of medium and large‐seeded species was decreased roughly 200% in response to biocrust presence (220%, Est = −0.698, *p* < 0.0001‐ and −180%, Est = −0.633, *p* < 0.0001 respectively; Figure 2, Table S2).

SEED_MASS also mediated the effects of biocrusts on emergence across different SOIL_REFERENCE_STATEs (i.e., the control substrate to which intact biocrusts are being compared in each study) When compared to emergence on filter paper, biocrusts increased the emergence of medium‐seeded species 700% (Est = 0.227, *p* < 0.0001; Figure S3, Table S2). Conversely, the emergence of large‐seeded species was decreased by half (−48.3%, Est = −0.286, *p* < 0.001; Figure S3, Table S2) while the emergence of small‐seeded species was unaffected on biocrust relative to filter paper controls. Interestingly, there was no significant effect of biocrust on emergence within any of the seed mass categories relative to bare soil controls (Table S2). However, relative to disturbed biocrust controls, medium and large‐seeded species both experienced decreased emergence (920%, Est = −0.545 *p* < 0.0001 and −930%, Est = −1.04 *p* < 0.0001 respectively; Figure S3, Table S2), while small‐seeded species experienced no effect. Finally, compared to biocrust removal controls, we found that the emergence of small‐seeded species was doubled on intact biocrust (Est = 0.121, *p* = 0.005; Figure S3, Table S2) while the emergence of medium and large‐seeded species decreased substantially (−780%, Est = −0.444, *p* < 0.001 and −430%, Est = −0.753 respectively; Figure S3, Table S2).

SEED_MASS also mediated the differential effects of biocrusts on the emergence of native versus non‐native plant species (PLANT_ORIGIN). Overall, emergence responses of small and large seeded species were similar in native versus non‐native plant species, while medium species displayed divergent responses depending on plant origin (Figure 3). Emergence of small seeded species was slightly increased if non‐native (+7.8%, Est = 0.116, *p* = 0.004; Figure 3, Table S2), while that of native species was unaffected. Medium seeds had the starkest difference. Medium‐seeded native species experienced increased emergence (+141%, Est = 0.366, *p* < 0.0001; Figure 3, Table S2), while non‐native species emergence decreased (−780%, Est = −0.837, *p* < 0.0001; Figure 3, Table S2). Large seeded species experienced decreased emergence regardless of plant origin, with native species experiencing a 400% decrease in emergence (Est = −0.326, *p* < 0.0001; Figure 3, Table S2), and non‐native species experiencing a 160% decrease in emergence on biocrust (Est = −0.176, *p* = 0.0001; Figure 3, Table S2).

#### 4.1.2 Seed Appendages Control the Effects of Biocrusts on Emergence for Different Biocrust Types and Native vs. Non‐Native Species

Seed appendages also mediated the effects of biocrusts on emergence across different biocrust types (Figures 4 and 6). Emergence of seeds with appendages was decreased on cyanobacteria dominated biocrusts 1500% (Est = −0.392, *p* < 0.0001; Figure 5, Table S2) while cyanobacteria dominated biocrusts had no effect on the emergence of seeds without an appendage. Lichen dominated crusts increased the emergence of seeds with appendages by 1000% (Est = 0.366, *p* < 0.0001) and decreased the emergence of seeds without an appendage by 900% (Est = −0.417, *p* < 0.0001, Figure 5; Table S2). Moss dominated crusts increased emergence of seeds with appendages by 1700% (Est = 0.581, *p* < 0.0001), but had no effect on seeds without an appendage (Figure 5, Table S2). Finally, mixed biocrust communities increased the emergence of seeds with appendages by +3600% (Est = 1.238, *p* < 0.0001) but had no effect on seeds without an appendage (Est = −0.0601, *p* = 0.557, Figure 5, Table S2).

The effects of PLANT_ORIGIN on emergence responses to biocrust also varied between seeds with and without appendages. Emergence of non‐native species lacking appendages decreased slightly in the presence of biocrusts (−18.8%, Est = −0.103, *p* = 0.001) while emergence of non‐native seeds with appendages was unaffected (Figure S4, Table S2). Overall, native species emergence was unaffected by biocrust presence regardless of whether seeds had appendages (Figure S4, Table S2).

#### 4.1.3 Seed Morphological Traits May Affect C4 Grass Interactions With Biocrusts, but Patterns Remain Unclear

PLANT_FUNCTIONAL_GROUP (i.e., C3 Grasses, C4 Grasses, Nitrogen‐fixing Forbs, Non‐Nitrogen‐Fixing Forbs, Nitrogen‐Fixing Woody Plants, and Non‐Nitrogen‐Fixing Woody Plants) influenced overall emergence responses to biocrust presence (Table S2). Among plant functional types, only C4 grasses overall experienced significant changes in emergence on biocrust (*p* = 0.0002, Table S2). While we aimed to explore whether morphological seed traits explained this pattern, due to small sample sizes for some plant functional groups (Table S1) and within subsets of interactions among functional traits of interest (e.g., PLANT_FUNCTIONAL_GROUP x SEED_MASS), we were unable to incorporate these interactions into our meta‐regression model to formally test our hypothesis that seed traits mediate the effects of biocrusts on the emergence of plants from different plant functional groups. Instead, we separately explored potential relationships among morphological seed traits and C4 grass responses to biocrust presence.

We qualitatively evaluated eight unique C4 grass species that occurred in one or more studies. Of the eight C4 grass species, five (62.5%) have seeds with appendages and six are native species. Four of the species have small seeds, one has medium seeds, and three have large seeds. These proportions were not reflected in the individual studies. The majority of the C4 grass studies have small seeds (S; 0.00–0.50 g/1000 seeds) with 71.1% of studies (*n* = 27, Table S1), while 10.0% have medium seeds (M; 0.51–1.00 g/1000 seeds; Table S1) and 18.4% have large seeds (L; 1.1+ g/1000 seeds; Table S1). Most C4 grass studies had seeds that lack appendages (68.4%, *n* = 26 studies, Table S1), while only 31.6% had appendages. The majority of C4 grass studies used native species (88.9%; *n* = 40, Table S1) while 11.1% were non‐native. When examining the interaction between C4 grasses and biocrust types, cyanobacteria‐dominated biocrusts made up 17.8% (*n* = 16) of studies, while moss‐dominated biocrusts made up 38.9% of studies (*n* = 35), lichen‐dominated biocrusts made up 21.1% of studies (*n* = 19) and mixed biocrust communities made up 22.2% of studies (*n* = 20). Collectively, unexpectedly, C4 grasses possessed traits that we found to promote emergence on biocrusts: C4 species included in the data set were generally small and had appendages. As such, while we might expect C4 grasses would experience increased emergence on biocrusts, this was not the case.

## 5 Discussion

Across global drylands, biocrusts are important ecosystem engineers (Belnap and Gardner 1993; Weber et al. 2022) and can have substantial impacts on plant recruitment (synthesized in Havrilla et al. 2019), though mechanisms underlying these interactions have remained unclear. Building upon a prior global meta‐analysis of emergence responses to biocrust presence (Havrilla et al. 2019), we used meta‐regression to analyze 321 published studies of plant emergence responses to biocrusts and explore the potential role of morphological seed traits in structuring responses. We found that seed traits interacted with biocrust characteristics in complex ways to determine emergence responses (Figure 6), and particularly, seed mass and structure (i.e., appendages) influenced the effects of biocrusts on plant emergence. First, seed mass interacted with biocrust community type, soil reference state, and plant origin to control the effects of biocrusts on emergence. Second, seed appendages differentially influenced emergence responses to biocrusts in native versus non‐native plant species and moderated the effects of different biocrust community types. In addition to these findings, we highlight key limitations and future research opportunities to increase understanding of biocrust–plant interactions using trait‐based frameworks.

### 5.1 Seed Mass and Appendages Influence Emergence Outcomes on Different Biocrust Community Types

Observed patterns in emergence responses to different biocrust community types suggest interactions between biocrust surface topography, seed mass, and seed morphological structure (i.e., appendages). Biocrust community types often differ in their morphological and physical (microtopographic) structure (Rosentreter et al. 2007; Colesie et al. 2016), characteristics that are often associated with climate and disturbance legacies (West 1990; Weber et al. 2022; Caster et al. 2021). For example, in hot climates or recently disturbed areas, biocrusts are often smooth, with low surface roughness and dominated by cyanobacteria with interspersed open patches of bare soil. In cooler ecosystems that experience freeze–thaw cycles and frost‐heaving (Chen et al. 2023) and at later stages of biocrust succession, biocrusts often form rolling microtopography and/or pinnacles dominated by cyanobacteria, lichens, and mosses reaching heights of 5–15 cm with few sections of open mineral soil surface (Rosentreter et al. 2007).

In our study, biocrust community types that typically have rougher surfaces (i.e., lichen, moss, and mixed communities, Rosentreter et al. 2007) decreased the emergence of large to medium‐seeded species. This could suggest that the rougher microtopography of these biocrusts may pose a barrier to larger seeds, as supported by Zhang et al. (2016) and Li et al. (2005). These rougher biocrusts may also elevate larger seeds above the mineral soil surface, increasing opportunities for seed predation by rodents and ants in situ (Dylewski et al. 2020, Maron et al. 2012; Pearson et al. 2014). Conversely, biocrusts with smoother microtopography (e.g., recently disturbed biocrusts or cyanobacteria dominated biocrusts) increased the emergence of large and medium seeds on cyanobacterial biocrusts. This result could be because cyanobacterial biocrusts are more likely to have smooth microtopography and exposed patches of bare soil, which could allow for more access to the mineral soil surface for seeds. Larger seeds, in particular, which have more stored resources to support emergence and growth (Baskin and Baskin 2014), in the absence of this physical barrier, might experience increased emergence in the presence of biocrust cyanobacteria, which tend to increase soil moisture (Eldridge et al. 2020). Copeland and McDonald (2012) stress the advantage of a larger seed size in dryland environments to have an increased chance of moisture uptake.

Interactive effects of seed appendages and biocrust community type on emergence may also be explained by seed interactions with biocrust surface microtopography (Zhang et al. 2016). Overall, emergence of seeds with appendages increased on lichen, moss, and mixed biocrust communities, which generally have coarser, more developed microtopography and more surface cracking (Rosentreter et al. 2007). This could suggest that coarser biocrusts might lift appendaged seeds and force them into the substrate, thereby increasing contact with the soil surface, or perhaps that they offer more favorable microsites and/or opportunities for seeds with appendages to be caught and/or retained within the biocrust surface until favorable conditions for emergence occur (Havrilla and Barger 2018; Zhang and Belnap 2015). In contrast, emergence of seeds with appendages was decreased on cyanobacterial biocrusts. This may be because early successional cyanobacterial biocrusts, or those in hotter environments, are often smoother with lower surface roughness. As such, there may be fewer opportunities for lift, catching, or favorable positioning of seeds with appendages within the mineral soil substrate or favorable microsites within the biocrust surface, which could leave seeds exposed to seed predation and/or desiccation on or above the soil surface (Song et al. 2020) and decreased emergence.

### 5.2 Seed Trait Structure Effects of Biocrusts on Native Versus Non‐Native Plant Emergence

Seed mass and appendages also controlled how the emergence of native versus non‐native plant species responded to biocrust presence. As in Havrilla et al. (2019), we found that biocrusts decreased the emergence of non‐native plant species but had neutral effects on native species overall, suggesting that biocrusts may act as a biotic control of non‐native plant invasion in drylands (Bowker et al. 2022; Havrilla and Barger 2018; Slate et al. 2019). Interestingly, our analyses showed that seed mass may partially explain these differential effects. While emergence responses of small (0.00–0.50 g/1000 seeds) and large (1.1+ g/1000 seeds) seeded species to biocrust presence were similar in native versus non‐native species (slightly positive in small seeds, negative for large seeds; Figure 3), responses of medium‐seeded species that weigh between 0.51–1.00 g/1000 seeds diverged dramatically between native versus non‐native species. Native medium‐seeded species experienced increased emergence, while non‐native medium‐seeded species experienced substantial decreases in emergence on biocrust. This result was surprising but could suggest that variation in seedling emergence responses to biocrusts is explained by other sources of variation at either end of the seed mass spectrum; at middling seed mass levels, effects of biocrusts on emergence diverge according to plant origin. Plants with medium seeds that coevolved in their native ranges alongside biocrusts may possess traits that are more suited to emergence on biocrust relative to non‐native species that may not be as well adapted (McTavish et al. 2021) and/or that biocrust microbes help alleviate dormancy and recruitment barriers to seeds with shared coevolutionary history (Eldridge et al. 2021).

Seed appendages also mediated the effects of biocrusts on native versus non‐native species emergence. While the emergence of native species was ambivalent to appendage presence, in non‐native species, biocrusts decreased the emergence of species without appendages but did not affect species with appendages. This result may suggest that while the emergence of non‐native species is, on average, more likely to be inhibited by biocrust presence, the presence of appendages (e.g., awns) may help some non‐native species overcome emergence barriers posed by biocrusts. Some plant species possessing awns may be specially adapted to promote seed movement and allow seeds to burrow into the soil surface. For example, some plant species possess hygroscopic awns, an adaptation that allows a seed to move across the surface into a favorable microsite (Peart 1979) or to “drill” into the soil when the awn is wetted (Briggs and Morgan 2011), which could aid seeds in overcoming physical barriers posed by biocrusts to plant emergence.

### 5.3 Study Limitations and Opportunities for Future Research

Our study demonstrates that basic morphological seed traits (e.g., mass, appendages) can be used to predict the effects of biocrusts on plant emergence but are limited in several ways. These limitations highlight opportunities to consider future research directions that could improve understanding of biocrust controls on plant emergence in drylands using functional trait‐based approaches.

1. *Availability and quality of seed trait data are limited. We call for expanded plant trait data reporting at large*. First, we relied on publicly available trait databases (e.g., TRY Plant database (Kattge et al. 2020)), KEW SID (Royal Botanic Gardens Kew 2020) to add seed traits to a prior database of studies of emergence responses to biocrusts (Havrilla et al. 2019). However, these queries ultimately resulted in an incomplete data set. For some plant species, there were incomplete seed trait records, and for others there were no morphological seed trait data available at all. We call for increased reporting of plant functional traits in ecological studies of plants, and measurement and sharing of more detailed and robust morphological seed trait information at large (Carta et al. 2024). Relatedly, while easily accessible, publicly available plant trait data represent pooled averages, and, in some cases, may be misaligned with seeds used in the published studies and with traits of local varieties and cultivars (Cordlandwehr et al. 2013). The recording of specific morphological seed trait data in studies of plant emergence responses to biocrusts would aid in the facilitation of future research that seeks to explore the species‐specific effects of biocrusts on plant emergence and recruitment.
2. *Opportunities for functional trait‐based studies of biocrusts are many*. In part due to the limited availability of seed trait data, in our study, we were only able to fully analyze the effects of two relatively simple and orthogonal morphological seed traits (i.e., seed mass and seed appendage) on plant emergence responses to biocrusts. Innumerable other morphological seed characteristics could potentially moderate these interactions (e.g., whether a seed is mucilaginous or has a hygroscopic awn (Zhang et al. 2016; Benard et al. 2019) and internal seed morphology (e.g., embryo size ratio, seed coat ratio, dispersal structures and mechanisms; Carta et al. 2024). Future studies should explore such additional morphological traits and their potential roles in structuring outcomes of biocrust–plant interactions.
3. *Further explorations of biocrust effects on different plant functional groups are needed*. Due to the limited sample size here, future studies should also more directly examine the relationships between plant functional groups, plant and seed traits, and biocrusts. Here, we explored potential links between seed traits and previously observed differences in emergence responses to biocrusts across different plant functional groups. We found that C4 grasses experienced overall decreased emergence on biocrusts, however we were unable to determine if morphological seed traits were responsible for this decrease due to limited sample sizes and trait information. Additional empirical studies should examine interactions between different plant functional types (e.g., C4 vs. C3 grasses; Havrilla et al. 2019), their traits, and biocrusts.
4. Consideration of the ever‐growing body of literature on biocrust–plant interactions offers opportunities for future research. Here, we only included effect sizes for studies of biocrust effects on plant emergence published between 1940 and 2017 included in the Havrilla et al. (2019) database. The body of research on this topic has since continued to grow (e.g., Bowker et al. 2022; Eldridge et al. 2021; Havrilla and Barger 2018; Hoose et al. 2022; Huber and Kollmann 2020; McIntyre et al. 2021; Slate et al. 2019; Song et al. 2020) and like those studies included in Havrilla et al. (2019), studies have continued to show variable effects of biocrusts on plant emergence. Future efforts exploring the effects of seed traits on biocrust–plant interaction outcomes can build upon this meta‐analysis to include more recent studies.
5. *Greater global representation of biocrust–plant interaction studies is needed—*Biocrusts can be found in every region of the globe, from the arid Southwestern United States to the polar regions of Antarctica, from sea level to the alpine (Weber et al. 2022). As discussed in Havrilla et al. (2019), our data set of published literature was geographically limited and lacked broad representation of studies from South America, Africa, Australia, and Polar regions. This limitation could be explained by the fact that (1) most published studies of biocrust–plant interactions were from regions within the northern hemisphere and the Global North. Further, (2) a more comprehensive multilingual data search process could have improved data selection and coverage (Zenni et al. 2023). The Havrilla et al. (2019) from which data for our meta‐analysis were extracted included only papers published or translated in English and Chinese. Incorporation of other language papers could lead to more equal geographic representation of studies across the globe, especially in South America, Africa, and other regions in the Global South.

### 5.4 Seed Traits Improve Predictions of Biocrust–Plant Interaction Outcomes in Drylands

Trait‐based approaches are increasingly used to understand patterns and mechanisms underlying plant emergence and community assembly processes (Funk et al. 2017; Larson et al. 2015; Saatkamp et al. 2019). Results from our study reveal that functional traits interact with biocrusts to structure plant emergence patterns across global drylands. Such knowledge increases understanding of dryland plant recruitment patterns and may have relevance for predicting outcomes of plant community assembly (HilleRisLambers et al. 2012; Levine et al. 2004; Lortie et al. 2004). This information may in turn assist land managers in planning conservation and restoration activities under changing climate and disturbance regimes in drylands. Biocrusts are declining worldwide because of climate change, aridification, and intensifying land degradation (Finger‐Higgens et al. 2022). As such, our results, combined with projections of changes in biocrust cover and composition, might be used to improve predictions of how biocrust presence (or absence) might affect dryland plant communities and plant regeneration in a changing climate. Finally, as land stewards in drylands prepare to adapt management to these challenges, new understanding of how seed traits mediate biocrust–plant interactions could be key to decision‐making about dryland conservation and restoration planning. For example, seed trait information may provide guides about which plant species may be successful in seed‐based restoration efforts in degraded systems where biocrust and plant communities are being restored in tandem.

## Author Contributions

**J. Bacovcin:** conceptualization (equal), data curation (lead), formal analysis (lead), visualization (lead), writing – original draft (lead), writing – review and editing (supporting). **C. McIntyre:** formal analysis (supporting), investigation (supporting), writing – review and editing (equal). **C. A. Havrilla:** conceptualization (equal), data curation (supporting), formal analysis (supporting), funding acquisition (lead), investigation (supporting), methodology (supporting), supervision (lead), visualization (supporting), writing – review and editing (equal).

## Conflicts of Interest

The authors declare no conflicts of interest.

## Supporting information


Data S1.

Appendix S1.

## Data Availability

All the required data are uploaded as Supporting Information.
