# Supplementary material for: Morphological Seed Traits Structure Relationships Between Biocrusts and Plant Emergence
Source: Ecol Evol. 2025 Jun 1;15(6):e71450. doi: 10.1002/ece3.71450 (PMC12127115; doi:10.1002/ece3.71450)
Supplement: Supplementary file 2 — Appendix S1. [file ECE3-15-e71450-s002.docx]

**APPENDIX**

Supporting results and information for Bacovcin et al. (2025).

**
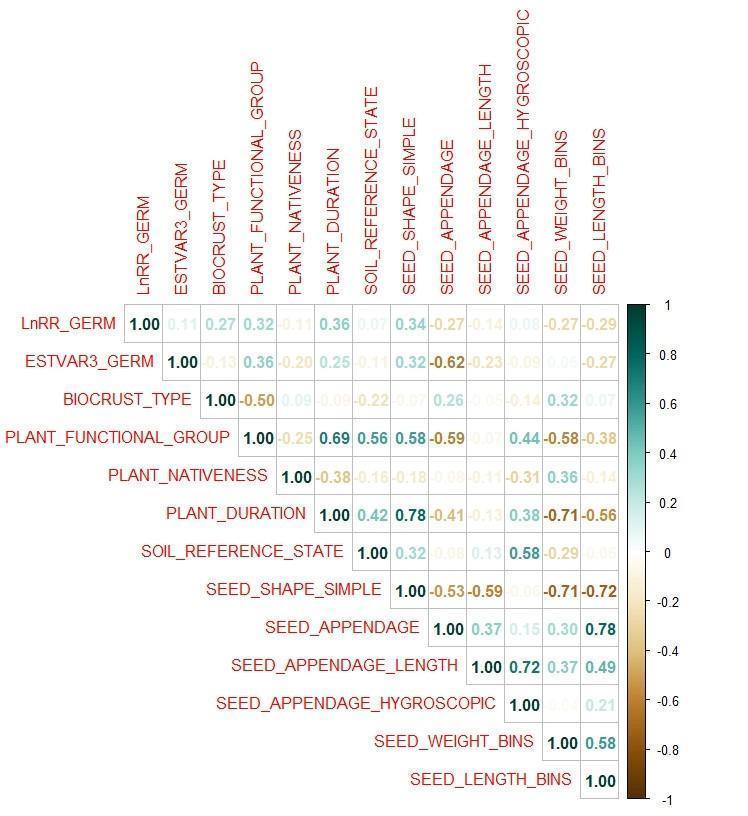
**

**Supplementary Figure 1.** Correlation matrix showing pearson correlation coefficients between candidate variables considered for inclusion in meta-regression models.

**
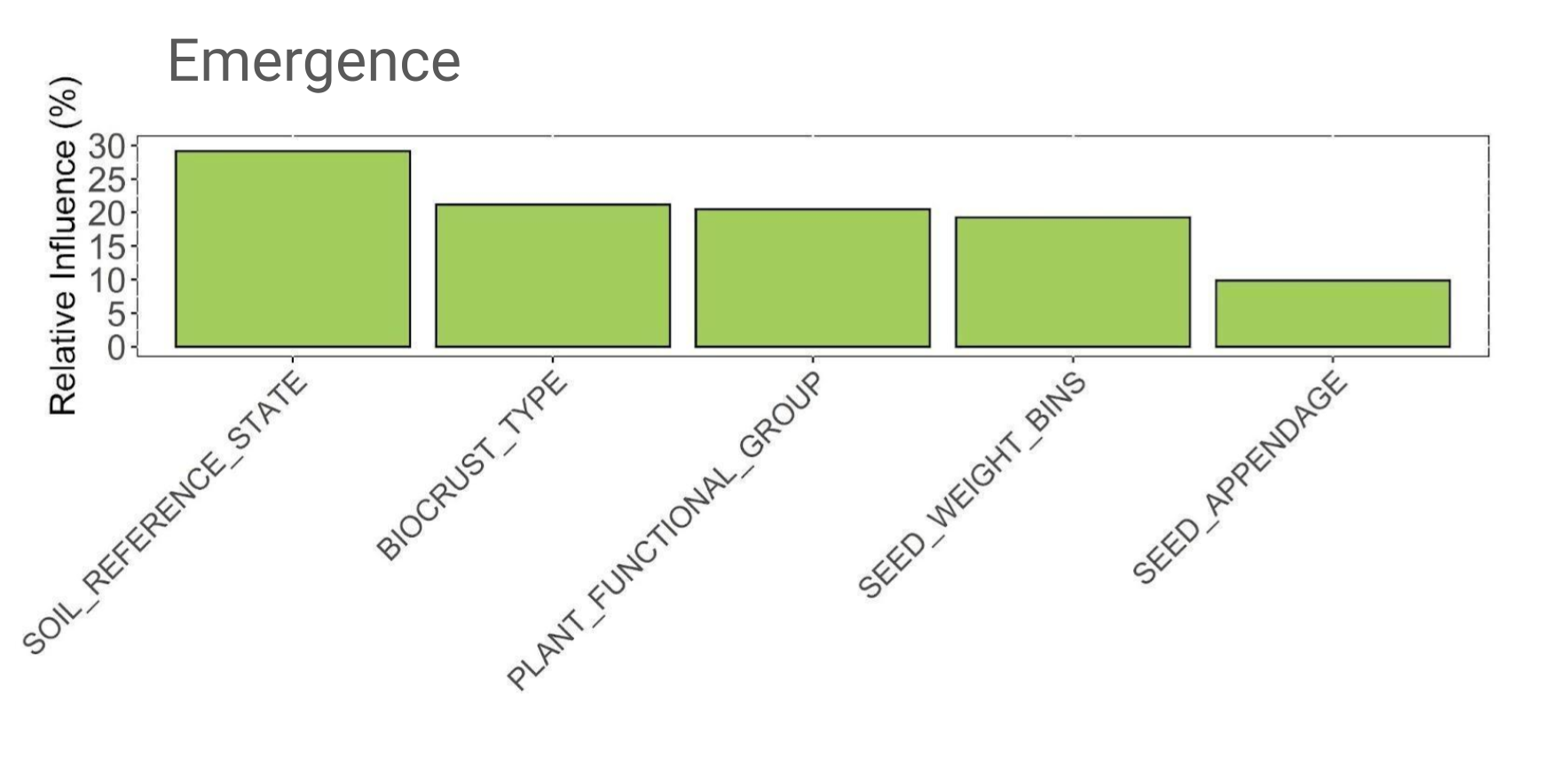
**

**Supplementary Figure 2.** Boosted regression tree model results showing the relative influence of variables on the log response of emergence to biocrust presence.


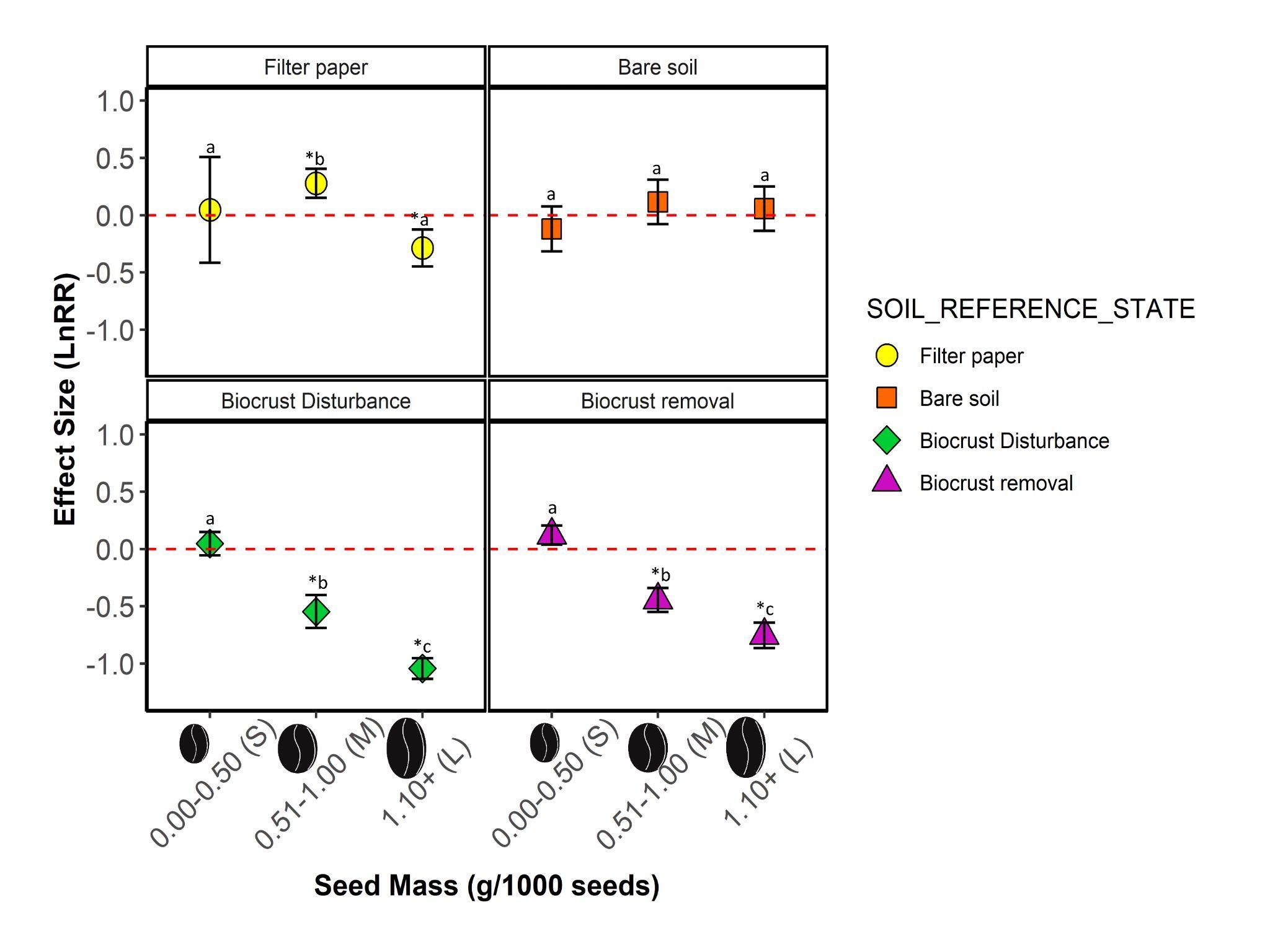


**Supplementary Figure 3.** Effect of seed mass on the log response ratio of emergence to biocrust across different soil reference states. Lower case letters (a-c) denote significantly significant pairwise differences while “*” indicates a significant difference from zero.


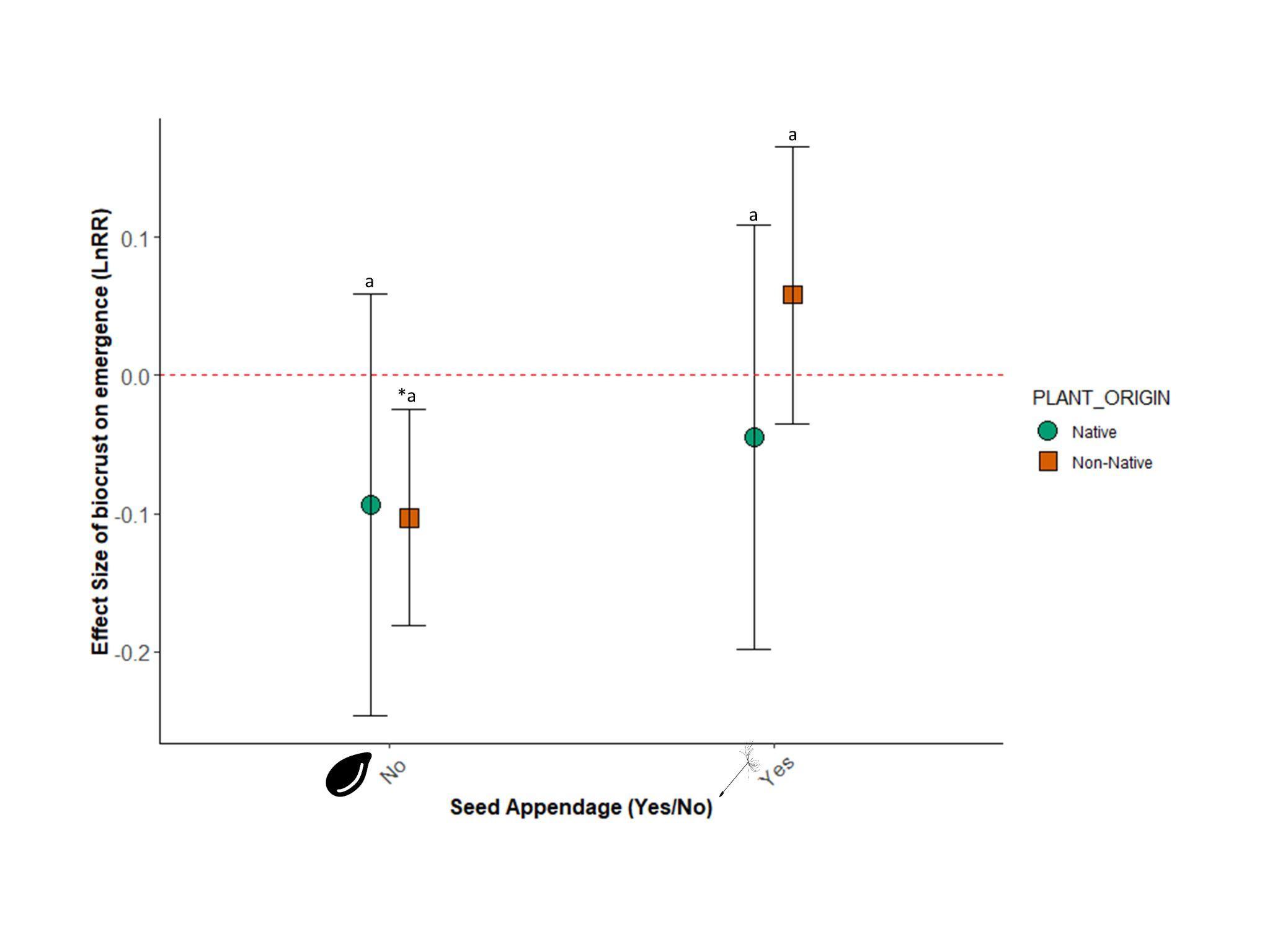


**Supplementary Figure 4.** Effect of seed appendage presence on the log response ratio of emergence to biocrust for native versus non-native plant species. Lower case letters (a-c) denote significantly significant pairwise differences while “*” indicates a significant difference from zero.

**Supplementary Table 1.** Number of studies (sample size) for each seed trait (e.g., seed mass, seed appendage presence, plant origin) and biocrust community type for studies of C4 grasses included in our database.

| **Variable** | **Sample Size** |
| --- | --- |
| **Seed Mass** | |
| Small (0-0.50 g/1000 seeds) | 27 |
| Medium (0.51-1.0 g/1000 seeds) | 4 |
| Large (1.1+ g/1000 seeds) | 7 |
| **Seed Appendage** | |
| No | 26 |
| Yes | 12 |
| **Plant Origin** | |
| Native | 40 |
| Non-native | 5 |
| **Biocrust Community Type** | |
| Cyanobacteria | 16 |
| Moss | 35 |
| Lichen | 19 |
| Mixed | 20 |

**Supplementary Table 2:** Results of the multivariate mixed-effects model that was used to analyze the candidate variables and their interactions.

| **Multivariate Model** | | | | | | | |
| --- | --- | --- | --- | --- | --- | --- | --- |
| Emergence Response | Fixed effects | Est | SE | z-value | p-value | ci.lb | ci.ub |
| *Intercept* | | 0.119 | 0.142 | 0.838 | 0.402 | -0.160 | 0.398 |
| **BIOCRUST_TYPE** | | | | | | | |
| Cyanobacteria | | -0.840 | 0.078 | -10.843 | **<.0001** | -0.992 | -0.688 |
| Lichen | | -0.383 | 0.041 | -9.363 | **<.0001** | -0.464 | -0.303 |
| Moss | | 0.0742 | 0.339 | 2.137 | **0.033** | 0.006 | 0.139 |
| Mixed | | 0.0939 | 0.045 | 2.091 | **0.037** | 0.00590 | 0.1820 |
| **PLANT_FUNCTIONAL_GROUP** | | | | | | | |
| C3 Grass | | 0.262 | 0.131 | 2.003 | **0.045** | 0.00570 | 0.519 |
| C4 Grass | | -0.262 | 0.131 | -2.003 | **0.045** | -0.519 | -0.00570 |
| N-fixing Forb | | -0.107 | 0.008 | -13.120 | **<.0001** | -0.123 | -0.0909 |
| N-fixing Woody Plant | | 0.700 | 0.066 | 10.585 | **<.0001** | 0.571 | 0.830 |
| Non-N-fixing Forb | | -0.0516 | 0.010 | -5.350 | **<.0001** | -0.0706 | -0.0327 |
| Non-N-Fixing Woody Plant | | 0.0937 | 0.047 | 2.180 | **0.013** | 0.0195 | 0.168 |
| **SOIL_REFERENCE_STATE** | | | | | | | |
| Filter Paper | | -0.0991 | 0.341 | -0.290 | 0.772 | -0.768 | 0.570 |
| Bare Soil | | 0.885 | 0.180 | 4.919 | **<.0001** | 0.532 | 1.237 |
| Biocrust Disturbance | | 0.0879 | 0.057 | 1.545 | 0.123 | -0.0237 | 0.200 |
| Biocrust Removal | | 0.102 | 0.047 | 2.391 | **0.029** | 0.0103 | 0.195 |
| **PLANT_ORIGIN** | | | | | | | |
| Native | | 0.735 | 0.056 | 13.114 | **<.0001** | 0.625 | 0.845 |
| Non-Native | | -0.176 | 0.063 | -2.790 | **0.005** | -0.300 | -0.0525 |
| **SEED_APPENDAGE** | | | | | | | |
| No | | -0.640 | 0.083 | -7.686 | **<.0001** | -0.803 | -0.477 |
| Yes | | -0.638 | 0.074 | -8.655 | **<.0001** | -0.782 | -0.493 |
| **SEED_MASS** | | | | | | | |
| 0.00-0.50 (S) | | 1.464 | 0.206 | 7.113 | **<.0001** | 1.0608 | 1.868 |
| 0.51-1.0 (M) | | 0.577 | 0.0493 | 11.688 | **<.0001** | 0.480 | 0.674 |
| 1.1+ (L) | | -0.247 | 0.0508 | -4.852 | **<.0001** | -0.346 | -0.147 |
| **Interactions** | | | | | | | |
| **PLANT_ORIGIN x SEED_MASS** | | | | | | | |
| Native x 0.00-0.5 (S) | | 0.107 | 0.084 | 1.280 | 0.201 | -0.0571 | 0.272 |
| Native x 0.51-1.0 (M) | | 0.366 | 0.085 | 4.316 | **<.0001** | 0.200 | 0.532 |
| Native x 1.1+ (L) | | -0.326 | 0.084 | -3.900 | **<.0001** | -0.490 | -0.162 |
| Non-Native x 0-0.5 (S) | | 0.116 | 0.040 | 2.902 | **0.004** | 0.0376 | 0.194 |
| Non-Native x 0.51-1.0 (M) | | -0.837 | 0.053 | -15.719 | **<.0001** | -0.941 | -0.733 |
| Non-Native x 1.1+ (L) | | -0.176 | 0.046 | -3.800 | **0.0001** | -0.267 | -0.0854 |
| **PLANT_ORIGIN x SEED_APPENDAGE** | | | | | | | |
| Native x Yes | | -0.0449 | 0.079 | -0.572 | 0.567 | -0.199 | 0.109 |
| Native x No | | -0.0936 | 0.078 | -1.202 | 0.229 | -0.246 | 0.0590 |
| Non-Native x Yes | | 0.0582 | 0.048 | 1.223 | 0.221 | -0.0350 | 0.151 |
| Non-Native x No | | -0.103 | 0.040 | -2.587 | **0.010** | -0.182 | -0.0250 |
| **BIOCRUST_TYPE x SEED_MASS** | | | | | | | |
| Cyanobacteria x 0-0.5 (S) | | -0.167 | 0.095 | -1.752 | 0.080 | -0.353 | 0.0198 |
| Cyanobacteria x 0.51-1.0 (M) | | 0.253 | 0.028 | 9.067 | **<.0001** | 0.198 | 0.308 |
| Cyanobacteria x 1.1+ (L) | | 0.197 | 0.028 | 6.997 | **<.0001** | 0.142 | 0.252 |
| Lichen x 0-0.5 (S) | | -0.393 | 0.097 | -4.065 | **<.0001** | -0.583 | -0.204 |
| Lichen x 0.51-1.0 (M) | | 0.0370 | 0.053 | 0.695 | 0.487 | -0.0673 | 0.141 |
| Lichen x 1.1+ (L) | | -0.701 | 0.042 | -16.622 | **<.0001** | -0.784 | -0.619 |
| Moss x 0.-0.5 (S) | | 0.0521 | 0.094 | 0.554 | 0.580 | -0.132 | 0.236 |
| Moss x 0.51-1.0 (M) | | -0.263 | 0.033 | -7.876 | **<.0001** | -0.328 | -0.197 |
| Moss x 1.1+ (L) | | -0.375 | 0.031 | -12.011 | **<.0001** | -0.437 | -0.314 |
| Mixed x 0-0.5 (S) | | 0.453 | 0.098 | 4.650 | **<.0001** | 0.262 | 0.644 |
| Mixed x 0.51-1.0 (M) | | -0.698 | 0.041 | -16.999 | **<.0001** | -0.778 | -0.617 |
| Mixed x 1.1+ (L) | | -0.633 | 0.039 | -16.050 | **<.0001** | -0.711 | -0.556 |
| **SOIL_REFERENCE_STATE x SEED_MASS** | | | | | | | |
| Filter Paper x 0-0.5 (S) | | 0.0464 | 0.235 | 0.197 | 0.844 | -0.414 | 0.507 |
| Filter Paper x 0.51-1.0 (M) | | 0.277 | 0.064 | 4.307 | **<.0001** | 0.151 | 0.403 |
| Filter Paper x 1.1+ (L) | | -0.286 | 0.082 | -3.471 | **<.0001** | -0.447 | -0.125 |
| Bare Soil x 0-0.5 (S) | | -0.120 | 0.099 | -1.206 | 0.228 | -0.315 | 0.0749 |
| Bare Soil x 0.51-1.0 (M) | | 0.115 | 0.099 | 1.166 | 0.243 | -0.0783 | 0.309 |
| Bare Soil x 1.1+ (L) | | 0.0571 | 0.099 | 0.578 | 0.563 | -0.136 | 0.251 |
| Biocrust Disturbance x 0-0.5 (S) | | 0.0475 | 0.052 | 0.915 | 0.360 | -0.0543 | 0.149 |
| Biocrust Disturbance x 0.51-1.0 (M) | | -0.545 | 0.073 | -7.431 | **<.0001** | -0.689 | -0.402 |
| Biocrust Disturbance x 1.1+ (L) | | -1.0415 | 0.046 | -22.466 | **<.0001** | -1.132 | -0.951 |
| Biocrust Removal x 0-0.5 (S) | | 0.121 | 0.043 | 2.808 | **0.005** | 0.0367 | 0.206 |
| Biocrust Removal x 0.51-1.0 (M) | | -0.444 | 0.053 | -8.365 | **<.0001** | -0.548 | -0.340 |
| Biocrust Removal x 1.1+ (L) | | -0.753 | 0.056 | -13.425 | **<.0001** | -0.862 | -0.643 |
| **BIOCRUST_TYPE x SEED_APPENDAGE** | | | | | | | |
| Cyanobacteria x No | | -0.0340 | 0.102 | -0.332 | 0.740 | -0.234 | 0.166 |
| Cyanobacteria x Yes | | -0.392 | 0.050 | -7.795 | **<.0001** | -0.491 | -0.294 |
| Lichen x No | | -0.417 | 0.105 | -3.987 | **<.0001** | -0.622 | -0.212 |
| Lichen x Yes | | 0.366 | 0.057 | 6.426 | **<.0001** | 0.254 | 0.477 |
| Moss x No | | -0.169 | 0.102 | -1.656 | 0.010 | -0.369 | 0.0311 |
| Moss x Yes | | 0.581 | 0.059 | 9.817 | **<.0001** | 0.465 | 0.696 |
| Mixed x No | | -0.0601 | 0.102 | -0.588 | 0.557 | -0.260 | 0.140 |
| Mixed x Yes | | 1.238 | 0.055 | 22.370 | **<.0001** | 1.130 | 1.347 |
